# 9-(3-Bromo-5-chloro-2-hy­droxy­phen­yl)-10-(2-hy­droxy­eth­yl)-1,2,3,4,5,6,7,8,9,10-deca­hydro­acridine-1,8-dione

**DOI:** 10.1107/S1600536812050222

**Published:** 2012-12-15

**Authors:** Shaaban K. Mohamed, Mehmet Akkurt, Peter N. Horton, Antar A. Abdelhamid, Mahmoud A. A. El Remaily

**Affiliations:** a Chemistry and Environmental Division, Manchester Metropolitan University, Manchester M1 5GD, England; b Chemistry Department, Faculty of Science, Minia University, El-Minia, Egypt; c Department of Physics, Faculty of Sciences, Erciyes University, 38039 Kayseri, Turkey; d School of Chemistry, University of Southampton, Highfield, Southampton SO17 1BJ, England; e Department of Chemistry, Sohag University, 82524 Sohag, Egypt

## Abstract

In the title compound, C_21_H_21_BrClNO_4_, the dihydro­pyridine ring adopts a flattened boat conformation. The 3-bromo-5-chloro-2-hy­droxy­phenyl ring forms a dihedral angles of 84.44 (7)° with the dihydro­pyridine mean plane. The mol­ecular conformation is stabilized by an intra­molecular O—H⋯O hydrogen bond, with an *S*(8) ring motif. In the crystal, O—H⋯O and C—H⋯O hydrogen bonds link the mol­ecules, forming a three-dimensional network.

## Related literature

For the synthesis and bioactivity of acridines, see, for example: Karolak-Wojciechowska *et al.* (1996 ▶). For related structures, see: Abdelhamid *et al.* (2011*a* ▶,*b* ▶); Mohamed *et al.* (2012 ▶); Guo *et al.* (2004 ▶); Sughanya & Sureshbabu (2012 ▶); Yogavel *et al.* (2005 ▶). For ring puckering parameters, see: Cremer & Pople (1975 ▶). For hydrogen-bond motifs, see: Bernstein *et al.* (1995 ▶).
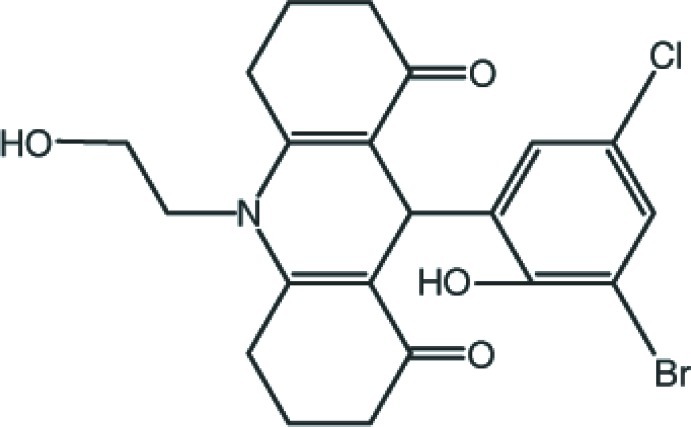


## Experimental

### 

#### Crystal data

C_21_H_21_BrClNO_4_
*M* *_r_* = 466.74
Monoclinic, 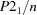

*a* = 8.810 (2) Å
*b* = 13.809 (3) Å
*c* = 15.797 (4) Å
β = 100.026 (4)°
*V* = 1892.5 (8) Å^3^
*Z* = 4
Mo *K*α radiation
μ = 2.34 mm^−1^
*T* = 100 K
0.22 × 0.14 × 0.03 mm

#### Data collection

Rigaku AFC12 (Right) diffractometer
Absorption correction: multi-scan (*CrystalClear-SM Expert*; Rigaku, 2012 ▶) *T*_min_ = 0.627, *T*_max_ = 0.933
14959 measured reflections
4299 independent reflections
4126 reflections with *I* > 2σ(i)
*R*_int_ = 0.024

#### Refinement

*R*[*F* ^2^ > 2σ(*F* ^2^)] = 0.028
*wR*(*F* ^2^) = 0.077
*S* = 1.03
4299 reflections
255 parameters
H-atom parameters constrained
Δρ_max_ = 0.86 e Å^−3^
Δρ_min_ = −0.38 e Å^−3^

### 

Data collection: *CrystalClear-SM Expert* (Rigaku, 2012 ▶); cell refinement: *CrystalClear-SM Expert*; data reduction: *CrystalClear-SM Expert* Expert; program(s) used to solve structure: *SHELXS97* (Sheldrick, 2008 ▶); program(s) used to refine structure: *SHELXL97* (Sheldrick, 2008 ▶); molecular graphics: *ORTEP-3 for Windows* (Farrugia, 2012 ▶); software used to prepare material for publication: *WinGX* (Farrugia, 2012 ▶) and *PLATON* (Spek, 2009 ▶).

## Supplementary Material

Crystal structure: contains datablock(s) global, I. DOI: 10.1107/S1600536812050222/hg5278sup1.cif

Structure factors: contains datablock(s) I. DOI: 10.1107/S1600536812050222/hg5278Isup2.hkl

Supplementary material file. DOI: 10.1107/S1600536812050222/hg5278Isup3.cml

Additional supplementary materials:  crystallographic information; 3D view; checkCIF report

## Figures and Tables

**Table 1:** Hydrogen-bond geometry (Å, °)

*D*—H⋯*A*	*D*—H	H⋯*A*	*D*⋯*A*	*D*—H⋯*A*
O1—H1*A*⋯O2	0.84	1.88	2.6749 (19)	158
O4—H4⋯O2^i^	0.84	1.94	2.782 (2)	176
C6—H6*B*⋯O3^ii^	0.99	2.33	3.051 (2)	129
C20—H20*A*⋯O3^ii^	0.99	2.53	3.486 (2)	163
C20—H20*B*⋯O1^i^	0.99	2.57	3.492 (2)	154

Symmetry codes: (i) 
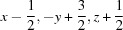
; (ii) 
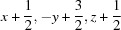
.

**Fig. 1.**
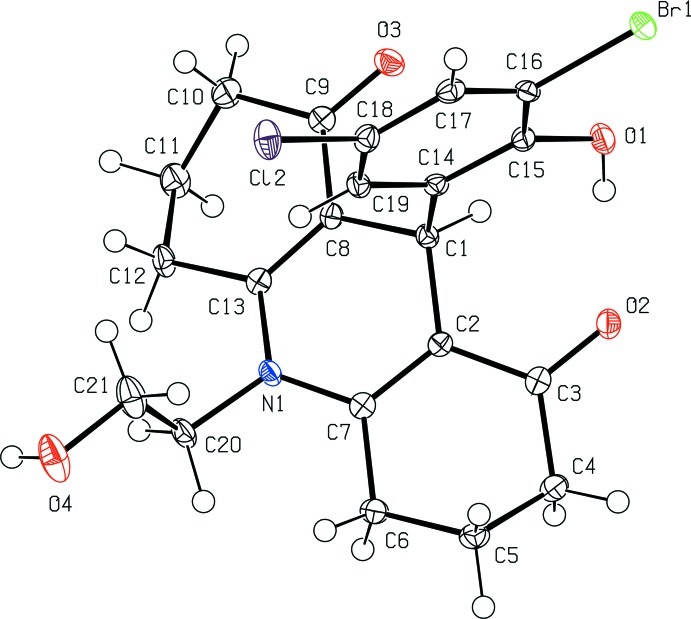
The title compound (I) with the atom numbering scheme. Displacement ellipsoids for non-H atoms are drawn at the 50% probability level.

**Fig. 2.**
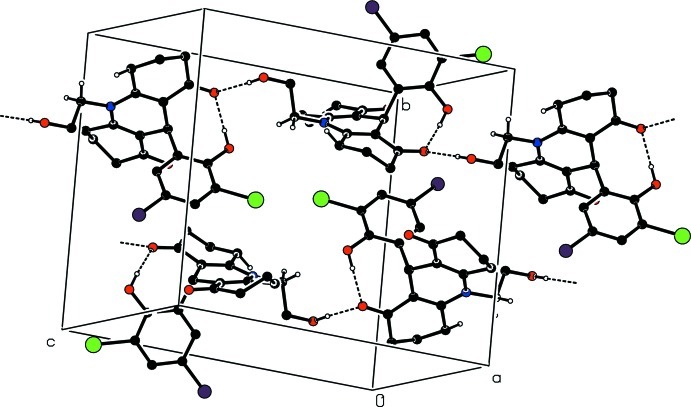
View of the packing and hydrogen bonding of (I) down the a axis. The hydrogen atoms not involved in the hydrogen bonds have been omitted for clarity.

## supplementary crystallographic information

### Comment

Acridine derivatives are one of the oldest and most successful classes of bioactive agents (Karolak-Wojciechowska *et al.*, 1996). Further to our on-going study on the synthesis and biological assessment of accridines (Mohamed *et al.*, 2012; Abdelhamid *et al.*, 2011**a*,b*), we report herein the synthesis and crystal structure determination of the title compound (I).

In the title compound (I), (Fig. 1), the dihydropyridine ring (N1/C1/C2/C7/C8/C13) is almost planar with a maximum deviation of 0.160 (2) Å for C1. The C14–C19 phenyl ring forms a dihedral angle of 84.44 (7)° with the dihydropyridine mean plane. In the 1,2,3,4,5,6,7,8,9,10-decahydroacridine ring system, the puckering parameters (Cremer & Pople, 1975) for the A(C2–C7), B(N1/C1/C2/C7/C8/C13) and C(C8–C13) rings are Q_T_ = 0.4695 (18) Å, θ = 121.6 (2) °, φ = 341.3 (2)° (for A); Q_T_ = 0.2607 (16) Å, θ = 77.7 (4) °, φ = 167.6 (4) ° (for B) and Q_T_ = 0.4511 (19) Å, θ = 126.1 (2) °, φ = 351.9 (3) ° (for C), respectively. The cyclohexenone rings A and C adopt sofa conformations, whereas the central ring B adopts flattened boat conformation. In (I), the bond lengths and angles are within normal ranges and and comparable with those in related similar compounds (Sughanya & Sureshbabu, 2012; Yogavel *et al.*, 2005; Guo *et al.*, 2004). The ethanol group is not coplanar with the attached 1,4-dihydropyridine ring, with a N1—C20—C21—O4 torsion angle of -174.31 (14)°.

The molecular conformation is stabilized by an intramolecular O—H···O hydrogen bond (Table 1), which forms a pseudo-eight-membered ring with graph set S(8) (Bernstein *et al.*, 1995).

In the crystal, molecules are linked by O—H···O and C—H···O hydrogen bonds, forming three dimensional network (Fig. 2, Table 1).

### Experimental

A mixture of 112 mg (0.001 mol) cyclohexane-1,3-dione, 236 mg (0.001 mol) 3-bromo-5-chloro-2-hydroxybenzaldehyde and 61 mg (0.001 mol) 2-aminoethanol in 50 ml ethanol was refluxed at 350 K and monitored by TLC till completion after 2 h. A mass solid precipitate was deposited on cooling, filtered and dried under vacuum then washed with cold ethanol and dried again.. The raw product was recrystallized from dimethyl formamide then triturated with ether to afford a good yield (73%) of high quality yellow plats (m.p. 483 K) that were suitable for X-ray difraction.

### Refinement

All H-atoms were placed in calculated positions with O—H = 0.84 Å, and C—H = 0.95 for aromatic, 0.99 for methylene and 1.00 Å for methine C—H = 0.97 Å for methylene *U*_iso_(H) = 1.2 *U*_eq_(C). They were refined using a riding model approximation with *U*_iso_(H) = 1.5*U*_eq_(O) for hydroxyl and *U*_iso_(H) = 1.2 *U*_eq_(C) for the other H atoms.

### Crystal data

**Table:**

C_21_H_21_BrClNO_4_	*F*(000) = 952
*M**_r_* = 466.74	*D*_x_ = 1.638 Mg m^−^^3^
Monoclinic, *P*2_1_/*n*	Mo *K*α radiation, λ = 0.71075 Å
Hall symbol: -P 2yn	Cell parameters from 4812 reflections
*a* = 8.810 (2) Å	θ = 2.5–27.5°
*b* = 13.809 (3) Å	µ = 2.34 mm^−^^1^
*c* = 15.797 (4) Å	*T* = 100 K
β = 100.026 (4)°	Plate, yellow
*V* = 1892.5 (8) Å^3^	0.22 × 0.14 × 0.03 mm
*Z* = 4	

### Data collection

**Table:**

Rigaku AFC12 (Right) diffractometer	4299 independent reflections
Radiation source: Rotating Anode	4126 reflections with *I* > 2σ(i)
Detector resolution: 28.5714 pixels mm^-1^	*R*_int_ = 0.024
profile data from ω–scans	θ_max_ = 27.5°, θ_min_ = 3.0°
Absorption correction: multi-scan (*CrystalClear-SM Expert*; Rigaku, 2012)	*h* = −10→11
*T*_min_ = 0.627, *T*_max_ = 0.933	*k* = −17→14
14959 measured reflections	*l* = −20→20

### Refinement

**Table:**

Refinement on *F*^2^	Primary atom site location: structure-invariant direct methods
Least-squares matrix: full	Secondary atom site location: difference Fourier map
*R*[*F*^2^ > 2σ(*F*^2^)] = 0.028	Hydrogen site location: inferred from neighbouring sites
*wR*(*F*^2^) = 0.077	H-atom parameters constrained
*S* = 1.03	*w* = 1/[σ^2^(*F*_o_^2^) + (0.0485*P*)^2^ + 1.1263*P*] where *P* = (*F*_o_^2^ + 2*F*_c_^2^)/3
4299 reflections	(Δ/σ)_max_ = 0.003
255 parameters	Δρ_max_ = 0.86 e Å^−^^3^
0 restraints	Δρ_min_ = −0.38 e Å^−^^3^

### Special details

**Table:**

Experimental. Rigaku *CrystalClear*-SM Expert 3.1 b5
Geometry. Bond distances, angles *etc*. have been calculated using the rounded fractional coordinates. All su's are estimated from the variances of the (full) variance-covariance matrix. The cell e.s.d.'s are taken into account in the estimation of distances, angles and torsion angles
Refinement. Refinement on *F*^2^ for ALL reflections except those flagged by the user for potential systematic errors. Weighted *R*-factors *wR* and all goodnesses of fit *S* are based on *F*^2^, conventional *R*-factors *R* are based on *F*, with *F* set to zero for negative *F*^2^. The observed criterion of *F*^2^ > σ(*F*^2^) is used only for calculating -*R*-factor-obs *etc*. and is not relevant to the choice of reflections for refinement. *R*-factors based on *F*^2^ are statistically about twice as large as those based on *F*, and *R*-factors based on ALL data will be even larger.

### Fractional atomic coordinates and isotropic or equivalent isotropic displacement parameters (Å^2^)

**Table:**

	*x*	*y*	*z*	*U*_iso_*/*U*_eq_	
Br1	1.02039 (2)	1.02608 (1)	0.11100 (1)	0.0178 (1)	
Cl2	0.89028 (5)	1.13026 (3)	0.42922 (3)	0.0206 (1)	
O1	0.85205 (14)	0.84648 (9)	0.14843 (8)	0.0171 (3)	
O2	0.91879 (13)	0.68133 (9)	0.23546 (7)	0.0155 (3)	
O3	0.44222 (13)	0.87300 (9)	0.18903 (8)	0.0175 (3)	
O4	0.69992 (16)	0.85677 (12)	0.68490 (9)	0.0294 (4)	
N1	0.65599 (15)	0.75304 (10)	0.46456 (8)	0.0125 (4)	
C1	0.69843 (17)	0.79777 (11)	0.29224 (10)	0.0113 (4)	
C2	0.79574 (17)	0.72563 (11)	0.34966 (10)	0.0114 (4)	
C3	0.90436 (17)	0.66869 (11)	0.31212 (10)	0.0124 (4)	
C4	0.99634 (19)	0.59201 (12)	0.36547 (11)	0.0169 (4)	
C5	1.03428 (19)	0.62450 (13)	0.45893 (11)	0.0165 (4)	
C6	0.88912 (18)	0.65080 (12)	0.49409 (11)	0.0152 (4)	
C7	0.77954 (17)	0.71276 (11)	0.43370 (10)	0.0121 (4)	
C8	0.54946 (17)	0.81352 (11)	0.32480 (10)	0.0114 (4)	
C9	0.42314 (18)	0.85498 (12)	0.26225 (10)	0.0134 (4)	
C10	0.27404 (19)	0.87759 (14)	0.29195 (11)	0.0202 (5)	
C11	0.24551 (19)	0.80920 (14)	0.36209 (11)	0.0203 (5)	
C12	0.38110 (18)	0.80674 (14)	0.43714 (11)	0.0165 (4)	
C13	0.53272 (17)	0.79239 (11)	0.40620 (10)	0.0122 (4)	
C14	0.78575 (17)	0.89267 (11)	0.28563 (10)	0.0113 (4)	
C15	0.85590 (17)	0.91091 (12)	0.21354 (10)	0.0128 (4)	
C16	0.93144 (18)	0.99934 (13)	0.20933 (10)	0.0135 (4)	
C17	0.94463 (17)	1.06742 (12)	0.27456 (11)	0.0143 (4)	
C18	0.87751 (19)	1.04633 (12)	0.34544 (11)	0.0146 (4)	
C19	0.79829 (18)	0.96063 (12)	0.35129 (10)	0.0131 (4)	
C20	0.65018 (19)	0.75423 (14)	0.55771 (10)	0.0168 (4)	
C21	0.6898 (2)	0.85513 (15)	0.59499 (12)	0.0242 (5)	
H1	0.67320	0.76910	0.23330	0.0140*	
H1A	0.85100	0.78990	0.16790	0.0260*	
H4	0.61310	0.84430	0.69740	0.0440*	
H4A	1.09300	0.57970	0.34330	0.0200*	
H4B	0.93660	0.53100	0.36150	0.0200*	
H5A	1.10350	0.68140	0.46340	0.0200*	
H5B	1.08930	0.57180	0.49410	0.0200*	
H6A	0.83570	0.59050	0.50590	0.0180*	
H6B	0.91900	0.68570	0.54920	0.0180*	
H10A	0.18800	0.87260	0.24270	0.0240*	
H10B	0.27710	0.94490	0.31370	0.0240*	
H11A	0.22750	0.74320	0.33800	0.0240*	
H11B	0.15140	0.82970	0.38350	0.0240*	
H12A	0.38450	0.86830	0.46960	0.0200*	
H12B	0.36580	0.75330	0.47660	0.0200*	
H17	0.99800	1.12660	0.27080	0.0170*	
H19	0.75240	0.94830	0.40040	0.0160*	
H20A	0.72430	0.70640	0.58780	0.0200*	
H20B	0.54570	0.73570	0.56680	0.0200*	
H21A	0.78940	0.87620	0.58030	0.0290*	
H21B	0.60970	0.90150	0.56860	0.0290*	

### Atomic displacement parameters (Å^2^)

**Table:**

	*U*^11^	*U*^22^	*U*^33^	*U*^12^	*U*^13^	*U*^23^
Br1	0.0204 (1)	0.0193 (1)	0.0158 (1)	0.0017 (1)	0.0089 (1)	0.0053 (1)
Cl2	0.0244 (2)	0.0190 (2)	0.0201 (2)	−0.0061 (2)	0.0086 (2)	−0.0083 (2)
O1	0.0224 (6)	0.0168 (6)	0.0136 (5)	−0.0002 (5)	0.0072 (5)	−0.0009 (5)
O2	0.0148 (5)	0.0180 (6)	0.0145 (5)	0.0010 (4)	0.0048 (4)	−0.0014 (5)
O3	0.0163 (6)	0.0228 (6)	0.0126 (5)	0.0012 (5)	0.0006 (4)	0.0024 (5)
O4	0.0222 (7)	0.0506 (9)	0.0163 (6)	−0.0094 (6)	0.0056 (5)	−0.0090 (6)
N1	0.0129 (6)	0.0163 (7)	0.0084 (6)	0.0000 (5)	0.0021 (5)	−0.0004 (5)
C1	0.0106 (7)	0.0136 (7)	0.0096 (7)	0.0000 (5)	0.0016 (5)	−0.0002 (6)
C2	0.0101 (7)	0.0114 (7)	0.0123 (7)	−0.0003 (5)	0.0011 (5)	−0.0008 (6)
C3	0.0107 (7)	0.0114 (7)	0.0150 (7)	−0.0021 (5)	0.0020 (5)	−0.0024 (6)
C4	0.0164 (7)	0.0155 (8)	0.0192 (8)	0.0035 (6)	0.0045 (6)	0.0019 (7)
C5	0.0138 (7)	0.0182 (8)	0.0168 (8)	0.0026 (6)	0.0005 (6)	0.0028 (6)
C6	0.0154 (7)	0.0158 (8)	0.0142 (7)	0.0012 (6)	0.0020 (6)	0.0026 (6)
C7	0.0106 (7)	0.0116 (7)	0.0137 (7)	−0.0021 (5)	0.0013 (5)	−0.0012 (6)
C8	0.0108 (7)	0.0117 (7)	0.0116 (7)	−0.0006 (5)	0.0020 (5)	−0.0017 (6)
C9	0.0126 (7)	0.0134 (7)	0.0136 (7)	−0.0010 (6)	0.0009 (6)	−0.0012 (6)
C10	0.0130 (7)	0.0288 (9)	0.0183 (8)	0.0048 (7)	0.0017 (6)	0.0013 (7)
C11	0.0134 (7)	0.0293 (9)	0.0182 (8)	−0.0011 (7)	0.0027 (6)	−0.0009 (7)
C12	0.0116 (7)	0.0262 (9)	0.0127 (7)	−0.0004 (6)	0.0047 (6)	−0.0018 (7)
C13	0.0110 (7)	0.0120 (7)	0.0132 (7)	−0.0010 (5)	0.0011 (6)	−0.0020 (6)
C14	0.0085 (6)	0.0134 (7)	0.0115 (7)	0.0013 (5)	0.0005 (5)	0.0018 (6)
C15	0.0105 (7)	0.0166 (8)	0.0110 (7)	0.0027 (6)	0.0012 (5)	−0.0001 (6)
C16	0.0114 (7)	0.0170 (7)	0.0126 (7)	0.0031 (6)	0.0038 (6)	0.0059 (6)
C17	0.0109 (7)	0.0141 (7)	0.0176 (8)	0.0011 (6)	0.0015 (6)	0.0025 (6)
C18	0.0138 (7)	0.0155 (7)	0.0142 (7)	0.0012 (6)	0.0015 (6)	−0.0029 (6)
C19	0.0114 (7)	0.0168 (8)	0.0114 (7)	0.0007 (6)	0.0029 (6)	0.0008 (6)
C20	0.0149 (7)	0.0271 (9)	0.0083 (7)	0.0013 (6)	0.0018 (6)	0.0002 (6)
C21	0.0209 (9)	0.0358 (11)	0.0163 (8)	−0.0070 (7)	0.0044 (7)	−0.0073 (8)

### Geometric parameters (Å, º)

**Table:**

Br1—C16	1.8939 (17)	C14—C19	1.389 (2)
Cl2—C18	1.7480 (18)	C14—C15	1.410 (2)
O1—C15	1.356 (2)	C15—C16	1.398 (2)
O2—C3	1.2517 (19)	C16—C17	1.385 (2)
O3—C9	1.223 (2)	C17—C18	1.385 (2)
O4—C21	1.408 (2)	C18—C19	1.385 (2)
O1—H1A	0.8400	C20—C21	1.529 (3)
O4—H4	0.8400	C1—H1	1.0000
N1—C20	1.481 (2)	C4—H4A	0.9900
N1—C7	1.384 (2)	C4—H4B	0.9900
N1—C13	1.406 (2)	C5—H5A	0.9900
C1—C14	1.532 (2)	C5—H5B	0.9900
C1—C2	1.510 (2)	C6—H6A	0.9900
C1—C8	1.506 (2)	C6—H6B	0.9900
C2—C3	1.443 (2)	C10—H10A	0.9900
C2—C7	1.371 (2)	C10—H10B	0.9900
C3—C4	1.500 (2)	C11—H11A	0.9900
C4—C5	1.524 (2)	C11—H11B	0.9900
C5—C6	1.524 (2)	C12—H12A	0.9900
C6—C7	1.501 (2)	C12—H12B	0.9900
C8—C13	1.351 (2)	C17—H17	0.9500
C8—C9	1.470 (2)	C19—H19	0.9500
C9—C10	1.503 (2)	C20—H20A	0.9900
C10—C11	1.510 (3)	C20—H20B	0.9900
C11—C12	1.530 (2)	C21—H21A	0.9900
C12—C13	1.513 (2)	C21—H21B	0.9900
			
C15—O1—H1A	109.00	C2—C1—H1	108.00
C21—O4—H4	109.00	C8—C1—H1	108.00
C13—N1—C20	119.40 (13)	C14—C1—H1	108.00
C7—N1—C20	121.30 (13)	C3—C4—H4A	110.00
C7—N1—C13	119.30 (13)	C3—C4—H4B	110.00
C2—C1—C14	111.47 (13)	C5—C4—H4A	110.00
C2—C1—C8	109.12 (13)	C5—C4—H4B	110.00
C8—C1—C14	112.17 (13)	H4A—C4—H4B	108.00
C1—C2—C7	121.55 (14)	C4—C5—H5A	109.00
C1—C2—C3	117.41 (13)	C4—C5—H5B	109.00
C3—C2—C7	121.02 (14)	C6—C5—H5A	109.00
O2—C3—C4	120.40 (14)	C6—C5—H5B	109.00
C2—C3—C4	119.06 (14)	H5A—C5—H5B	108.00
O2—C3—C2	120.52 (14)	C5—C6—H6A	109.00
C3—C4—C5	110.03 (14)	C5—C6—H6B	109.00
C4—C5—C6	111.52 (14)	C7—C6—H6A	109.00
C5—C6—C7	113.07 (14)	C7—C6—H6B	109.00
C2—C7—C6	121.69 (14)	H6A—C6—H6B	108.00
N1—C7—C6	117.60 (13)	C9—C10—H10A	109.00
N1—C7—C2	120.64 (14)	C9—C10—H10B	109.00
C1—C8—C13	122.65 (14)	C11—C10—H10A	109.00
C9—C8—C13	121.91 (14)	C11—C10—H10B	109.00
C1—C8—C9	115.43 (13)	H10A—C10—H10B	108.00
C8—C9—C10	118.16 (14)	C10—C11—H11A	109.00
O3—C9—C8	120.16 (15)	C10—C11—H11B	109.00
O3—C9—C10	121.64 (15)	C12—C11—H11A	109.00
C9—C10—C11	111.44 (15)	C12—C11—H11B	109.00
C10—C11—C12	112.04 (14)	H11A—C11—H11B	108.00
C11—C12—C13	111.51 (14)	C11—C12—H12A	109.00
N1—C13—C8	120.51 (14)	C11—C12—H12B	109.00
N1—C13—C12	117.61 (13)	C13—C12—H12A	109.00
C8—C13—C12	121.85 (14)	C13—C12—H12B	109.00
C1—C14—C15	120.25 (14)	H12A—C12—H12B	108.00
C15—C14—C19	119.57 (14)	C16—C17—H17	121.00
C1—C14—C19	120.17 (14)	C18—C17—H17	121.00
O1—C15—C16	119.10 (14)	C14—C19—H19	120.00
C14—C15—C16	118.28 (14)	C18—C19—H19	120.00
O1—C15—C14	122.61 (14)	N1—C20—H20A	110.00
C15—C16—C17	122.46 (15)	N1—C20—H20B	110.00
Br1—C16—C15	118.54 (12)	C21—C20—H20A	110.00
Br1—C16—C17	119.00 (13)	C21—C20—H20B	110.00
C16—C17—C18	117.80 (15)	H20A—C20—H20B	108.00
Cl2—C18—C19	118.86 (13)	O4—C21—H21A	109.00
C17—C18—C19	121.64 (15)	O4—C21—H21B	109.00
Cl2—C18—C17	119.50 (13)	C20—C21—H21A	109.00
C14—C19—C18	120.20 (15)	C20—C21—H21B	109.00
N1—C20—C21	110.34 (14)	H21A—C21—H21B	108.00
O4—C21—C20	111.92 (16)		
			
C7—N1—C13—C12	161.29 (14)	C5—C6—C7—C2	12.9 (2)
C7—N1—C20—C21	103.01 (17)	C5—C6—C7—N1	−170.23 (14)
C13—N1—C20—C21	−77.86 (18)	C1—C8—C13—C12	−178.67 (15)
C7—N1—C13—C8	−16.8 (2)	C9—C8—C13—N1	−179.81 (14)
C13—N1—C7—C2	11.4 (2)	C1—C8—C9—O3	1.0 (2)
C20—N1—C7—C2	−169.52 (15)	C1—C8—C9—C10	−176.73 (14)
C20—N1—C13—C12	−17.9 (2)	C13—C8—C9—O3	−179.82 (15)
C20—N1—C13—C8	164.11 (15)	C9—C8—C13—C12	2.2 (2)
C20—N1—C7—C6	13.6 (2)	C13—C8—C9—C10	2.4 (2)
C13—N1—C7—C6	−165.53 (14)	C1—C8—C13—N1	−0.7 (2)
C14—C1—C2—C7	98.95 (17)	O3—C9—C10—C11	151.65 (16)
C8—C1—C14—C15	−137.57 (15)	C8—C9—C10—C11	−30.6 (2)
C2—C1—C8—C13	20.3 (2)	C9—C10—C11—C12	54.0 (2)
C14—C1—C8—C9	75.41 (17)	C10—C11—C12—C13	−49.4 (2)
C2—C1—C8—C9	−160.57 (13)	C11—C12—C13—N1	−156.62 (15)
C2—C1—C14—C15	99.74 (17)	C11—C12—C13—C8	21.4 (2)
C2—C1—C14—C19	−79.27 (18)	C1—C14—C15—O1	−1.0 (2)
C8—C1—C14—C19	43.4 (2)	C19—C14—C15—C16	−2.4 (2)
C8—C1—C2—C7	−25.5 (2)	C1—C14—C19—C18	179.85 (15)
C14—C1—C2—C3	−82.55 (17)	C15—C14—C19—C18	0.8 (2)
C14—C1—C8—C13	−103.74 (17)	C1—C14—C15—C16	178.62 (14)
C8—C1—C2—C3	153.02 (13)	C19—C14—C15—O1	177.99 (15)
C1—C2—C7—C6	−172.02 (14)	C14—C15—C16—Br1	−178.05 (12)
C1—C2—C3—O2	1.8 (2)	O1—C15—C16—Br1	1.6 (2)
C7—C2—C3—C4	2.2 (2)	O1—C15—C16—C17	−177.82 (15)
C1—C2—C7—N1	11.2 (2)	C14—C15—C16—C17	2.5 (2)
C7—C2—C3—O2	−179.65 (15)	C15—C16—C17—C18	−1.1 (2)
C3—C2—C7—C6	9.5 (2)	Br1—C16—C17—C18	179.52 (12)
C1—C2—C3—C4	−176.32 (14)	C16—C17—C18—Cl2	−179.70 (13)
C3—C2—C7—N1	−167.22 (14)	C16—C17—C18—C19	−0.6 (2)
C2—C3—C4—C5	−34.7 (2)	C17—C18—C19—C14	0.7 (3)
O2—C3—C4—C5	147.20 (15)	Cl2—C18—C19—C14	179.80 (12)
C3—C4—C5—C6	55.54 (18)	N1—C20—C21—O4	−174.31 (14)
C4—C5—C6—C7	−45.49 (19)		

### Hydrogen-bond geometry (Å, º)

**Table:**

*D*—H···*A*	*D*—H	H···*A*	*D*···*A*	*D*—H···*A*
O1—H1*A*···O2	0.84	1.88	2.6749 (19)	158
O4—H4···O2^i^	0.84	1.94	2.782 (2)	176
C1—H1···O1	1.00	2.48	2.918 (2)	106
C6—H6*B*···O3^ii^	0.99	2.33	3.051 (2)	129
C20—H20*A*···O3^ii^	0.99	2.53	3.486 (2)	163
C20—H20*B*···O1^i^	0.99	2.57	3.492 (2)	154

Symmetry codes: (i) *x*−1/2, −*y*+3/2, *z*+1/2; (ii) *x*+1/2, −*y*+3/2, *z*+1/2.
